# Pleistocene refugia and genetic diversity patterns in West Africa: Insights from the liana *Chasmanthera dependens* (Menispermaceae)

**DOI:** 10.1371/journal.pone.0170511

**Published:** 2017-03-16

**Authors:** Andrew Chibuzor Iloh, Marco Schmidt, Alexandra Nora Muellner-Riehl, Oluwatoyin Temitayo Ogundipe, Juraj Paule

**Affiliations:** 1 Senckenberg Biodiversity and Climate Research Centre (BiK-F), Senckenberganlage 25, Frankfurt am Main, Germany; 2 Department of Botany and Molecular Evolution, Senckenberg Research Institute and Natural History Museum Frankfurt, Senckenberganlage 25, Frankfurt am Main, Germany; 3 Department of Molecular Evolution and Plant Systematics & Herbarium LZ, Institute of Biology, Leipzig University, Johannisallee 21–23, Leipzig, Germany; 4 German Centre for Integrative Biodiversity Research (iDiv) Halle-Jena-Leipzig, Deutscher Platz 5e, Leipzig, Germany; 5 Department of Botany, Molecular Systematics Laboratory, University of Lagos, Akoka, Lagos, Nigeria; National Cheng Kung University, TAIWAN

## Abstract

Processes shaping the African Guineo-Congolian rain forest, especially in the West African part, are not well understood. Recent molecular studies, based mainly on forest tree species, confirmed the previously proposed division of the western African Guineo-Congolian rain forest into Upper Guinea (UG) and Lower Guinea (LG) separated by the Dahomey Gap (DG). Here we studied nine populations in the area of the DG and the borders of LG and UG of the widespread liana species, *Chasmanthera dependens* (Menispermaceae) by amplified fragment length polymorphism (AFLP), a chloroplast DNA sequence marker, and modelled the distribution based on current as well as paleoclimatic data (Holocene Climate Optimum, ca. 6 kyr BP and Last Glacial Maximum, ca. 22 kyr BP). Current population genetic structure and geographical pattern of cpDNA was related to present as well as historical modelled distributions. Results from this study show that past historical factors played an important role in shaping the distribution of *C*. *dependens* across West Africa. The Cameroon Volcanic Line seems to represent a barrier for gene flow in the present as well as in the past. Distribution modelling proposed refugia in the Dahomey Gap, supported also by higher genetic diversity. This is in contrast with the phylogeographic patterns observed in several rainforest tree species and could be explained by either diverging or more relaxed ecological requirements of this liana species.

## Introduction

The African Guineo-Congolian rain forest is the second largest block of rain forest on Earth with about 6400 endemic plant species [1], and considered a biodiversity hotspot [2]. Repeated fragmentation of the tropical forest was suggested due to climate oscillations for the last one million years [3, 4]. Based on White’s chorological analyses [5], the African Guineo-Congolian rain forest can be divided into three phytogeographic units: Upper Guinea (UG), Lower Guinea (LG) and Congolia. All three units are characterized by considerable historical contractions, shifts and/or expansions [4]. Thus, current ranges of species or particular lineages are defined by the location of their refugia during the Last Glacial Maximum (LGM) as well as by postglacial migration routes [6].

Phylogenetic and population genetic studies provide valuable data to test the forest refuge theory as well as infer the location of refugia in Africa [7]. These studies have been particularly insightful for tree species due to their longevity, high reproductive output, but low speciation rates [8]. Comparative phylogeographic analyses of trees from LG and Congolia revealed a partial congruence of phylogeographic patterns with LGM forest refugia proposed by Maley [9–15]. Interestingly, phylogeographic patterns congruent with those of tree species were also found for Marantaceae herbs and lianas in this region [16].

The split between UG and LG rain forest is mainly constituted by a savanna corridor in Benin, Togo and eastern Ghana, also referred to as the Dahomey Gap (DG), and is caused by current rainfall gradients [14, 17–19]. Nevertheless, the two forest blocks were probably for the last time connected during the Holocene Humid Period (ca. 6–9 thousand years before present (kyr BP) [20] and several rain forest plant species are still present in the DG but scattered in microhabitats. It is therefore worthy of note that the forest species in the DG may either originate due to recent migrations from the main forest blocks (UG, LG), or constitute a remnant of the last period of rain forest connection. Interestingly, a recent phylogeographic study on the tree species *Distemonanthus benthamianus* (Fabaceae) [19] indicated that the history of the DG populations are consistent with paleo-vegetation data suggesting that the forest flora of the DG might be a relic of the early Holocene period when the Guineo-Congolian forest reached its maximum geographical distribution.

Lianas (woody vines) are non-self-supporting plants that use the architecture of trees to ascend to the forest canopy [21]. They play an important role in forest dynamics accomplishing various key indicator properties (i.e. gap-phase dynamics, transpiration and carbon sequestration). Lianas are particularly abundant and diverse in lowland tropical forests, where they constitute up to 40% of the woody biomass and more than 25% of the woody species [21], and contribute substantially to the forest leaf area [22, 23]. Interestingly, Martin *et al*. [24] report that they are more prevalent in areas of secondary forest succession and are often able to compete effectively against tree and shrub species under acute and chronic disturbance. For lianas, it is expected that their genotypic diversity, in comparison to trees, mirrors younger historical events due to presumably shorter life cycles [25, 26] and that the current genetic patterns might be more structured due to smaller dispersal distances in the tropical understory [27].

*Chasmanthera dependens* is a dioecious forest liana of the family Menispermaceae. It is widely distributed from Sierra Leone eastwards to Eritrea and Somalia, and southwards through eastern DR Congo and Tanzania to Angola and Zambia [28]. *Chasmanthera dependens* occurs in dense evergreen and semi-deciduous humid forest, in gallery forest, in termite mound thickets, thalwegs, humid secondary forest and bush fallow, at low to medium altitudes (up to 1500 m). It has a preference for well drained soils in localities with good availability of water and light [29, 30]. The species is widely used in traditional medicine due to its contents of bitterns and alkaloids [30–32].

In this study, we sampled populations of *C*. *dependens* from the area of the DG and the borders of LG and UG, genotyped them with amplified fragment length polymorphism (AFLP), employed a chloroplast (cp) DNA sequence marker, and modelled the distribution based on current as well as paleoclimatic data (Holocene Climate Optimum, HCO, ca. 6 kyr BP and Last Glacial Maximum, LGM, ca. 21 kyr BP) in order to answer the following questions:

1. Was the distribution of *C*. *dependens* across West Africa influenced by past climatic changes (Pleistocene)? Which areas are indicated as LGM refugia using distributional models based on past climatic scenarios?
2. Which areas could be considered as LGM refugia based on patterns of genetic diversity? Are the patterns recovered by nuclear (AFLP) and chloroplast markers congruent and correspond to the postulated refugia indicated by distribution models?
3. Did the Dahomey Gap impact the present distribution of genetic diversity in this species? Is it possible to identify two diverging gene pools corresponding to refugia in UG and LG or is the genetic diversity distributed continuously?
4. Are the phylogeographic patterns of a liana congruent with generally postulated patterns for tree species?

## Materials and methods

### Plant material

Fresh leaf tissue of *C*. *dependens* was collected from five West African countries (Benin, Cameroon, Ghana, Nigeria and Togo) covering the area of the eastern UG, western LG as well as the DG. In total, 139 individuals representing nine populations were investigated with 7–39 individuals per population (Table 1, S1 Table). Samples collected within a 50 km radius were considered a population. At least one herbarium specimen was prepared from each locality. Herbarium specimens were deposited at the Herbarium Senckenbergianum (FR) as well as at the University of Lagos Herbarium (LUH). The coordinates for the field-collected material were obtained using a handheld GPS unit, and for all kinds of the geographical presentation, ArcView-ArcGIS v10.1 (ESRI, USA) was used.

**Table 1:** Sampling localities of studied *Chasmanthera dependens* populations.

Pop	Locality	Nb	Latitude/Longitude
BN01	Lama Forest, Benin	23	6.974/2.133
CMR01	Ngoro, Cameroon	13	4.878/11.351
CMR02	Mount Febé, Cameroon	9	3.915/11.494
GH01	Seya Breku, Ghana	10	5.487/-0.534
NG01	Nsukka, Nigeria	18	6.706/7.462
NG02	Obinze, Nigeria	39	5.403/6.968
NG03	Okeigbo, Nigeria	7	7.218/4.675
NG04	Ibadan, Nigeria	9	7.388/3.992
TG01	Anagali Forest, Togo	11	6.517/1.358

Pop–population code, Nb–number of studied samples.

### DNA extraction, PCR amplification and sequencing

Total genomic DNA was extracted from silica gel‐dried leaf tissue. Extraction of total genomic DNA followed the CTAB procedure of Doyle and Doyle [33], with the following modifications: 700 μl of CTAB buffer were used for initial incubation, 500 μl of isopropanol were used for DNA precipitation, with two subsequent washing steps using 100 μl of 70% ethanol each. Finally, DNA was dissolved in 200 μl 1 × TE including 2μl RNase (10 mg·ml^-1^). Alternatively, DNA was extracted with the QiagenDNeasy® Plant Mini Kit (Hilden, Germany) or the NucleoSpin Plant II Kit (Macherey-Nagel, Düren, Germany) from leaf fragments of approximately 1 cm^2^ size, following the manufacturers’ protocols. Extracted DNA was dissolved in 200 μl TE buffer and 2 units (U) RNase (10 mg·ml^-1^) were added.

The cpDNA trnH-psbA intergenic spacer was amplified using the primers trnH(gug) 5’-CGC GCA TGG TGG ATT CAC AAT CC-3’ and psbA 5’-GTT ATG CAT GAA CGT AAT GCT C-3’ [34]. The reaction mix of 25 μl contained 21.9 μl 1.1 × ReddyMix^TM^ PCR Master Mix (ThermoFisher Scientific, Waltham, USA), 0.5 μl bovine serum albumin (10 mg·ml^-1^) (New England BioLabs, Ipswich, USA), 1 μL dimethyl sulfoxide (DMSO; Carl Roth, Karlsruhe, Germany), 1 μl of template DNA, and 0.3 μL of each primer (10 μM). PCR reactions were performed on a Mastercycler® pro (Eppendorf, Hamburg, Germany), with initial denaturation of 2 min at 95 °C, followed by 35 cycles of denaturation at 95 °C for 1 min, annealing at 53 °C for 1 min and extension at 72 °C for 1 min, followed by a final extension step for 10 min at 72 °C. PCR products were cleaned using the NucleoSpin® Extract II Kit (Macherey-Nagel, Düren, Germany), or the QIAquick® Gel Extraction Kit (Qiagen, Hilden, Germany). Sequencing was accomplished for both strands using 3730 DNA analyzer (Applied Biosystems, Foster City, USA) by the laboratory centre of the Senckenberg Biodiversity and Climate Research Centre (BiK-F) with the primers used for PCR. Sequences were manually edited for bad quality bases and assembled in contigs using Geneious Pro v5.6.6 (Biomatters, Auckland, New Zealand). Sequences were aligned using the pairwise alignment algorithm implemented in Geneious Pro and the alignment was manually refined.

### Amplified fragment length polymorphism (AFLP) analysis

For a subset of 54 individuals plus 22 duplicate samples, AFLP analysis was performed using the protocol established by Vos *et al*. [35], with minor modifications: Approximately 300 ng of DNA was digested and ligated in a 15 μl reaction mix containing 1 × T4-ligase buffer and 1 × ATP solution (Bioline, London, UK), 50 mM NaCl, 0.75 μg BSA, 1.5 U T4-ligase (Bioline), 1 U MseI and 5 U EcoRI (New England Biolabs), and 0.37μM of EcoRI-adapter and 3.67 μM of MseI-adapter. The reaction mix was incubated at 37 °C for 3 h, followed by an inactivation step at 65 °C for 10 min. The restriction-ligation product was subsequently diluted ten-fold. For the pre‐selective PCR reaction, 2.5 μl of the diluted restriction‐ligation product were used in a total reaction volume of 12.5 μl which contained 10 × PCR buffer II (Applied Biosystems), 2 mM MgCl_2_, 0.8 mM dNTP mix, 0.2 μM EcoRI‐A primer (5’‐GACTGCGTACCAATTCA‐A‐3’), 0.2 μM MseI‐C primer (5’‐GATGAGTCCTGAGTAAC‐C‐3’) and 0.25 U AmpliTaq polymerase (Applied Biosystems). The reactions were held at 72 °C for 2 min, followed by 20 cycles of 94 °C for 20 s, 56 °C for 30 s, and 72 °C for 2 min, with a final 30 s extension at 60 °C, and were subsequently diluted ten-fold. For selective PCR, 2.5 μl of the diluted pre‐selective PCR product were used as a template in a total reaction volume of 12.5 μl. The PCR master mix contained 1 × GoldTaq buffer (Applied Biosystems), 2.5 mM MgCl_2_, 0.8mM dNTP mix, 0.2μM Mse primer, 0.08μM EcoRI fluorescence‐labeled primer (EcoRI‐ACG (NED)/MseI‐CTC, EcoRI‐AAG(6‐FAM)/MseI‐CTA, EcoRI‐AGC(VIC)/MseI‐CTG,EcoRI‐AGG(NED)/MseI‐CAT; EcoRI‐AAC(6‐FAM)/MseI‐CAG and EcoRI‐ACC(VIC)/MseI‐CTC), and 0.5 U AmpliTaq Gold (Applied Biosystems). The reactions were held at 95 °C for 5 min, followed by 13 cycles at 94 °C for 30 s, a touch down cycle of 65 °C to 56 °C (–0.7 °C per cycle) for 1 min and 72 °C for 1 min, followed by another 23 cycles at 94 °C for 30 s, 56 °C for 1 min and 72 °C for 1.5 min, with a final 8 min extension at 72 °C. Differentially fluorescence-labeled PCR products and GS600 LIZ size standards (Applied Biosystems) were multiplexed, and fragments were separated on a 3730 DNA Analyzer (Applied Biosystems). In each run, a total of 96 samples were analyzed, including one negative control and several other repeats (altogether 37%), as recommended by Bonin *et al*. [36]. Raw data were visualized and the fragments manually scored using GeneMarker v1.97 (Soft Genetics, State College, USA). Processed data were exported as a presence/absence matrix.

### Data analyses

Indels in the cpDNA sequences were manually coded for presence and absence using the approach described by Simmons and Ochoterena [37], and treated as single polymorphic sites. A statistical parsimony network among cpDNA haplotypes was reconstructed using TCS v1.2 [38] with a default connection limit of 95%. Haplotypes were then plotted as pie charts on the map of West Africa using the compiled site co-ordinates to show the distribution of haplotypes. Haplotype diversity (h) [39] and nucleotide diversity (π) [40] of populations were calculated using MEGA v5 [41] and DnaSP v5.10.1 [42].

For the AFLP dataset several statistical parameters such as total number of fragments, proportion of polymorphic fragments, number of private fragments, and Nei’s gene diversity for the whole sampling as well as for particular populations [40] were computed using the R‐script AFLPdat [43]. Main trends in genetic variation among individual genotypes were visualized by principal coordinate analysis based on Jaccard distances (PCoA) calculated using PASTv2.7 [44].

For both, cpDNA and AFLP datasets, F-statistics, AMOVA and Mantel tests (based on pairwise population F_ST_ matrix) were calculated in Arlequin v3.1 [45], and the significance value tested using a nonparametric permutation test following the method of Excoffier *et al*. [46].

### Distribution modelling

In order to investigate a relation of current genetic patterns to past processes, which might have shaped them, the potential distribution of *Chasmanthera dependens* was modelled using current and past climatic data. Occurrence records were compiled from several databases, including GBIF [47], the African Plant Database [48], and a record from Gnoumou *et al*. [49]. Doubles and doubtful records were removed (S2 Table), leaving a total of 131 georeferenced distribution points. Bioclimatic grids at a spatial resolution of 10’ were downloaded for the present as well as the LGM (ca. 22 kyr BP) and the HCO (ca. 6 kyr BP) from the WorldClim v1.4 database [50] and clipped to an extent covering tropical Africa. For projections into the past, we used WorldClim’s paleoclimate layers for CCSM4 and MPI-ESM-P global climate models. LGM and HCO were the periods in which the climate changed most abruptly in the recent past and the patterns recovered by the models could help us to trace footprints in the genetic variation. Highly correlated variables (absolute correlation coefficients higher than 0.8, S3 Table) and variables with implausible discontinuities were removed, leaving a set of six variables, that were used in Maxent v3.3.3 [51] for distribution models of *C*. *dependens* during the present and the LGM (Bio1 = Annual Mean Temperature, Bio6 = Min Temperature of Coldest Month, Bio7 = Temperature Annual Range, Bio12 = Annual Precipitation, Bio14 = Precipitation of Driest Month, Bio15 = Precipitation Seasonality). We removed duplicate records, reserved 25% of the occurrence points for testing, chose a number of 10,000 random background points (i.e. pseudoabsences), disabled hinge and threshold features in Maxent and used the median out of 100 model runs. For evaluation of the distribution models, we used the AUC (area under the model’s receiver-operator-characteristic curve) [52].

## Results

### Chloroplast DNA data and haplotype distribution

The cpDNA sequences were obtained for 139 individuals (Electronic Appendix 1). The length of the analyzed trnH-psbA fragments ranged from 244 to 256 bp. Nine nucleotide substitutions, one indel and two repeated sequence motifs were detected. The length of the alignment was 256 bp. After manual coding of the indels and removal of the repeated sequence motifs, the total length of the alignment was reduced to 244 bp, and 10 parsimony-informative sites were considered. Newly generated sequences were deposited in the GenBank (KX863354-KX863492, www.ncbi.nlm.nih.gov/genbank/).

**Fig 1:**
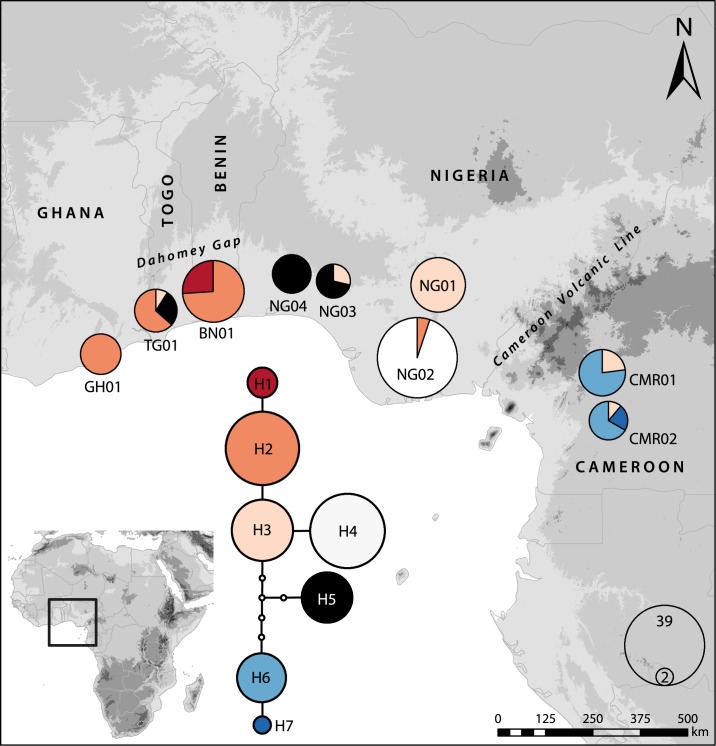
Statistical parsimony network based on trnH-psbA cpDNA sequences of *Chasmanthera dependens* and distribution of the cpDNA haplotypes in Western Africa. Small empty circles represent haplotypes that are not present, but necessary to link all the haplotypes recorded to the network. All haplotypes are separated from the nearest haplotype by one mutation/indel.

Seven haplotypes were identified, and the unrooted statistical parsimony haplotype network revealed three informal groups of haplotypes (Fig 1), separated from each other by four to five mutations. The first group consisted of four haplotypes (H1–H4), the second group of one haplotype (H5), and the third group of two haplotypes (H6–H7). Haplotypes H6 and H7 were exclusive to Cameroon populations, H4 was found only in Nigerian population NG02, and H1 only in Benin. In contrast, haplotype H2 was distributed in Nigeria, Benin, Togo and Ghana, and H3 was found throughout the whole studied area. Haplotype and nucleotide diversities of the populations and broader geographical units are summarized in Table 2. F-statistics and AMOVA results are summarized in Table 3. The highest values for haplotype and nucleotide diversities were recorded in populations from Cameroon (CMR02) and Togo (TG01).

**Table 2:** Indices of haplotypic (cpDNA) and genotypic (AFLP) diversity of *Chasmanthera dependens* populations.

	cpDNA				AFLP				
	Nb	Nb_haplo_	*h* ± SD	*π* [%] ± SD	Nb	FT	FP [%]	FPP	*D*_*g*_
**Total**	139	7	0.808 ± 0.013	0.00999 ± 0.00067	54	374	99.73	-	0.22
**PopBN01**	23	2	0.443 ± 0.080	0.00181 ± 0.00033	-	-	-	-	-
**PopCMR01**	13	2	0.385 ± 0.132	0.00631 ± 0.00217	12	275	69.25	23	0.22
**PopCMR02**	9	3	0.556 ± 0.165	0.00524 ± 0.00269	7	225	57.75	4	0.21
**PopGH01**	10	1	0.000	0.00000	9	217	53.48	14	0.20
**PopNG01**	18	1	0.000	0.00000	16	267	70.32	18	0.20
**PopNG02**	39	2	0.100 ± 0.063	0.00082 ± 0.00052	-	-	-	-	-
**PopNG03**	7	2	0.476 ± 0.171	0.00585 ± 0.00211	-	-	-	-	-
**PopNG04**	9	1	0.000	0.00000	-	-	-	-	-
**PopTG01**	11	3	0.564 ± 0.134	0.00745 ± 0.00197	10	235	61.23	12	0.22

Nb, number of individuals; Nb_haplo_, number of haplotypes; *h*, haplotype diversity [39]; *π*, nucleotide diversity [40];

SD, standard deviation; FT, total number of bands; FP, proportion of polymorphic bands; FPP, number of private bands and Nei’s genotypic diversity (*D*_*g*_).

**Table 3:** Analyses of molecular variance (AMOVAs) for cpDNA and AFLP data in *Chasmanthera dependens*.

Source of variation	d.f.	Sum of squares	Variance components	Percentage of variation	F_ST_
**cpDNA**					
**Among populations**	8	131.732	1.101	79.73	
**Within populations**	130	36.383	0.27987	20.27	
**Total**	138	168.115	1.38103		0.797
**AFLP**					
**Among populations**	4	268.671	2.663	6.39	
**Within populations**	49	1910.236	38.98441	93.61	
**Total**	53	2178.907	41.64741		0.064

### AFLP data analyses

**Fig 2:**
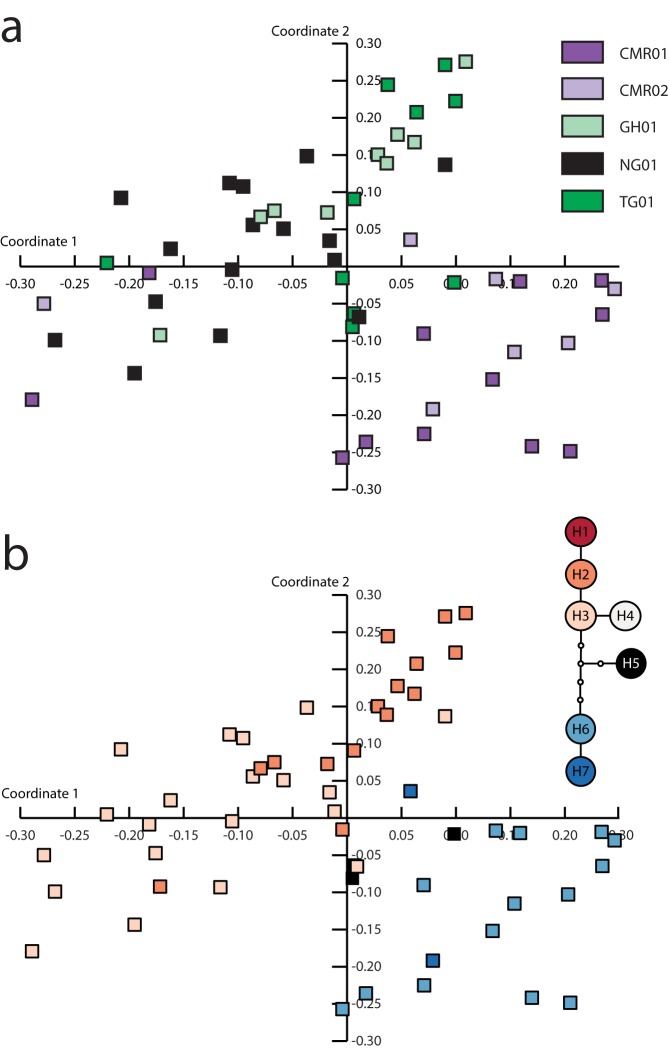
Principal co-ordinate analysis (PCoA) of AFLP genotypes of 54 samples of *Chasmanthera dependens* using Jaccard distances. The first two axes explained 9.27% and 7.33% of the total variation. Color-coding differentiates a) the populations and b) the haplotypes revealed by the statistical parsimony network analysis.

After removing fragments with an error rate of more than 15%, 374 clearly scorable fragments sized from 100 to 591bp were considered for further analyses, out of which 89.01% were polymorphic (Table 2, S4 Table). The repeatability (technical difference rate) [36] of replicated individuals was 89.83–97.16% (mean 93.65%). Two dimensional PCoA based on Jaccard distances separated populations from Nigeria, Benin, Togo and Ghana from Cameroon populations (Fig 2A). The separation also strongly reflected the division suggested by the haplotype network (Fig 2B). However, only 16.6% of the overall variation was explained by the first two axes.

The cpDNA and AFLP datasets revealed strikingly contrasting results, suggesting high population differentiation considering the cpDNA data (F_ST_ = 0.797), and very low population differentiation regarding the AFLP data (F_ST_ = 0.064) (Table 3). Mantel tests of both datasets proposed a weak (cpDNA) to strong (AFLP) correlation between matrices of genetic and geographic distances of populations (r_M_ = 0.373, p = 0.015, r_M_ = 0.623, p = 0.037, respectively).

### Species distribution modelling

**Fig 3:**
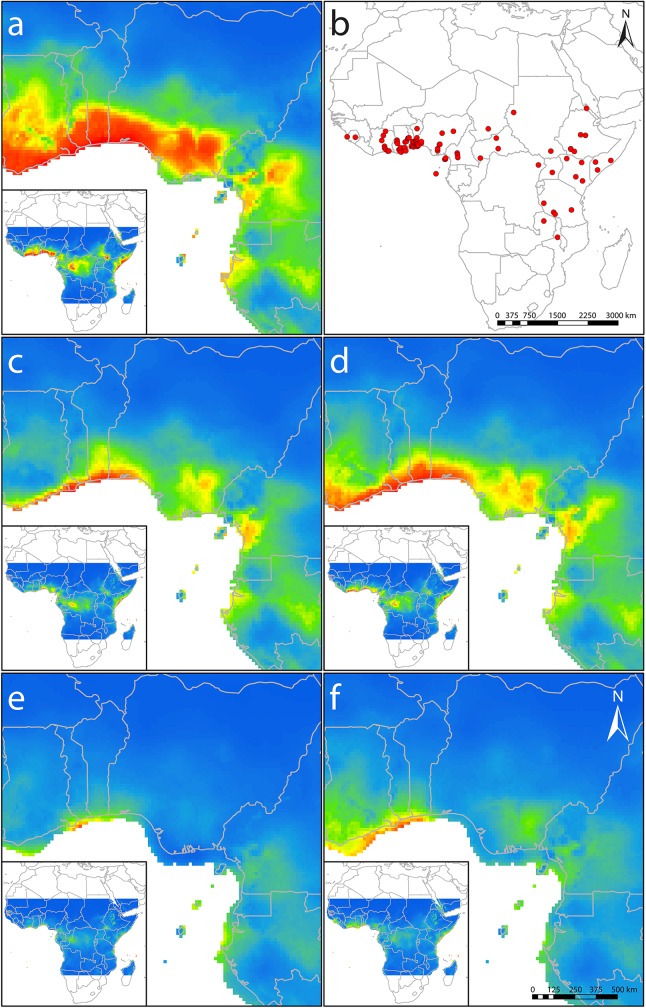
Current and historical species distribution models for *Chasmanthera dependens* in West Africa and tropical Africa, respectively. Probability of occurrence is represented by different colors from low (blue) to high (red). Results are based on the data from CCSM4 and MPI ESM-P paleoclimatic models representing the Last Glacial Maximum (LGM, ca. 21 kyr BP) and Holocene Climate Optimum (HCO, ca. 6 kyr BP), as well as current climate observations. (a) Model of current distribution; (b) red dots indicate current occurrence points, which served as a basis for modelling, (c) HCO, CCSM4; (d) HCO, MPI ESM-P; (e) LGM, CCSM4; (f) LGM, MPI ESM-P.

Present models reflect well the distribution known from occurrence records and literature (Fig 3A and 3B), apart from the localities in Tanzania, Zambia and Malawi. All single model runs had test AUC values above 0.7 with an average of 0.83. The bioclimatic variable with the highest contribution to the models was the minimum temperature of the coldest month (Bio6, 77.8%), followed by the temperature annual range (Bio7, 16.2%) and annual mean temperature (Bio1, 3.3%). The annual precipitation (Bio12, 2.7%) had the smallest contribution.

Beyond the known distributions, high probabilities of occurrence were also predicted for coastal Kenya and Tanzania. Distribution ranges for present, HCO and LGM, using both climate models, consistently showed a gap in the area of the Cameroon Volcanic Line (CVL), including Mt. Cameroon and the Bamenda Highlands (as well as westwards towards the Niger Delta). Furthermore, during the LGM the distribution range seems to have been much more fragmented in West Central Africa and the East African Rift zone than both nowadays or during the HCO. Interestingly, high distribution probabilities during the LGM were assigned to the coastal areas of Ghana, Togo and Benin, also referred to as the Dahomey Gap.

## Discussion

Geographic patterns of genetic diversity and differentiation of the African liana *Chasmanthera dependens* were investigated in this study in order to assess phylogeographic processes in West Africa using a descriptive genetic and distribution modelling approach. Particular focus of the modelling approach was given to populations representing the UG and LG phytogeographical units, and processes possibly accounting for observed patterns are discussed. For the distribution models climate grids at 1 km resolution were used, which are considered well-suited to account for the subcontinental extent of the study area and the objective of modelling past distributions. Details on the extent of microhabitat patches with possibly diverging microclimate were therefore not considered, which may lead to overestimations in the drier parts of the species range.

### Nuclear and cpDNA genetic differentiation

AFLP data for *Chasmanthera dependens* populations showed very low levels of genetic differentiation among the populations (F_ST_ = 0.064). Low genetic differentiation and high gene flow between populations can result from long-distance gene dispersal either by pollen or by seed [53]. However, significantly higher chloroplast genetic differentiation (F_ST_ = 0.797) suggests much higher pollen-mediated gene flow than gene flow by seed dispersal.

In tropical woody plants, pollen-mediated gene flow is thought to be more extensive than gene flow by seed [54, 55]. Animal-dispersed pollen can move over several kilometers in a continuous tropical forest [56] and wind-dispersed pollen probably over much longer distances. *Chasmanthera dependens* is a dioecious species with small greenish-yellow male flowers and small brownish female flowers in pseudo-racemose inflorescences and relatively large fleshy seeds. Hence, higher pollen-mediated gene flow in *C*. *dependens* could be explained by occasional wind pollination over long distances. On the other hand, fleshy seeds might also be considered an efficient strategy for moving seeds over certain distances [57], most probably by birds [58]. Nevertheless, pollination and seed dispersal agents of *C*. *dependens* as well as other climbers are still insufficiently documented [59]. In dioecious taxa, gender distribution and sex ratio are also strongly influencing gene flow [60]. Outcrossing mating system results in reduced population differentiation as reflected by the largely nuclear AFLPs, but bi-parental inbreeding also remains a possibility [61]. Moreover, small number of individuals of one sex can significantly reduce effective population size [62]. Hence, stochastic neutral processes and genetic drift can certainly contribute to population differentiation as reflected by the cp DNA data, considering also low population densities and patchy distribution pattern (A.C. Iloh—personal observation).

### Genetic divergence related to past climate fluctuations

Current geographical patterns of genetic diversity provide useful insights into species’ histories [63, 64], in particular if the current observations are combined with distribution modelling based on past climatic conditions. In our study, one haplotype was recovered throughout the whole studied area (H3, Fig 1). The presence of one haplotype suggests either past continuous distribution throughout the area or could be the result of dispersal events. As chloroplast haplotypes represent the seed parent [65], and our genetic data suggest that seed dispersal is limited, we consider past continuous distribution more likely. Distribution modelling suggested several gaps in the distribution within the study area, including the CVL, for at least 22 kyr. We therefore assume that haplotype H3 might represent a widespread ancestral haplotype spread throughout the distribution range in the moistest phase of the Eemian Interglacial period (125–120 kyr BP), the last period of continuous rainforest before the LGM, or even sooner [66].

Apart from haplotype H3, we identified two gene pools using both types of molecular markers (Figs 1 and 2) with a significant geographical pattern (Mantel tests). This pattern, however, does not correspond to the division of the proposed phytogeographic units (UG, LG), even though UG is under-represented in our sampling. Chloroplast markers revealed a distinct position of the Cameroon populations, having a set of unique haplotypes (H6, H7) and simultaneously having one of the highest nucleotide and haplotype diversities (Table 2). The differentiation of the Cameroonian populations in the cpDNA was also reflected in the AFLP analysis (Fig 2). The remainder of the West African populations could be considered a second gene pool constituted mainly by haplotypes H1‒H5. The Cameroon Volcanic Line (CVL) seems to represent a barrier between these gene pools, both today and in the past (Fig 3). Hence, we did not recover a gene pool differentiation corresponding to UG and LG, as observed in the legume tree species *Distemonanthus benthamianus* [19], but rather between Cameroon and the remainder of the West African populations. A specific genepool in the area of the DG in comparison to populations from Cameroon was also recovered in the rainforest tree *Symphonia globulifera* (Clusiaceae) [67] as well as in the dioecious tree *Milicia excelsa* (Moraceae) [12]. However, due to lack of sampling no relation to populations from Nigeria was elucidated. Contrariwise, one continuous genepool of the gallery forest legume tree *Erythrophleum suaveolens* (Fabaceae) was recovered throughout the UG and DG, reaching up to the CVL [68].

On the one hand, this finding supports the presence of refugia in Cameroon, which has also been previously suggested based on high genetic diversity documented in several tree species [10] and is also mirrored by higher probabilities using paleodistribution modelling (Fig 3C‒3F). On the other hand, we observed a certain west-east gradient in haplotype and nucleotide diversity in the second gene pool for populations from Ghana, Togo, Benin and Nigeria, revealing populations from Togo and Benin (TG01, BN01) as the genetically most diverse ones. Interestingly, Togo and Benin are representing the areas of dry vegetation (i.e., the DG), separating UG and LG, and higher haplotype diversity and uniform gene flow across the DG (haplotype H2, H3; Fig 2) is rather surprising. In order to explain this pattern, several scenarios could be assumed: 1) high haplotype diversity and haplotype endemism indicate a refugium at the locality or close by; 2) the locality might have been colonized from different refugia; or 3) the high diversity is a result of recent dispersal events. Dispersal events can be considered less likely due to low seed dispersal suggested by the comparison of cpDNA and AFLP markers (see discussion above). For the differentiation between the first two scenarios, distribution modelling and the presence of derived endemic haplotype H1 can provide valuable insights, even though our data provide only limited resolution and drier parts of the species range might be overestimated. It is remarkable that predicted distribution areas with highest probabilities in the models under LGM paleoclimatic scenarios are localized in the area of the DG (Fig 3E and 3F) from where *C*. *dependens* expanded during the HCO (Fig 3C and 3D). This implies that currently observed high diversity in the area might be very likely an outcome of LGM climatic fluctuations, and high haplotype and nucleotide diversity of the population in Togo (TG01, Table 2) and the presence of the haplotype H1 in the population BN01 (Fig 1) might reflect the presence of a LGM refugium as suggested by paleodistribution modelling (Fig 3). Alternatively, refugia might have been located further east in the UG phylogeographic unit, and BN01 and TG01 represent a melting pot of widely distributed haplotypes, which unfortunately cannot be tested with our sampling.

*Chasmanthera dependens* nowadays seems to be associated with dense evergreen and semi-deciduous humid forest. However, the species also occurs in gallery forest, in termite mound thickets, thalwegs, and bush fallow. Lianas are also generally considered to be more prevalent in areas of secondary forest succession and are often able to compete effectively against tree and shrub species under disturbed environmental conditions [24]. Based on the genetic data and distribution models, *C*. *dependens* seems not to be strictly associated with tropical rainforest, which might explain why the genetic patterns and distribution modelling do not reflect the UG/LG phylogeographic division. Gallery forests, disturbed forest habitats, and forest edges are currently present throughout savannas, and some of these habitats were most probably also present in the area of the DG during the LGM. Interestingly, evergreen and semi-deciduous rain forest is proposed during the LGM for most of current Nigeria based on paleovegetation data [69], and LGM paleoclimatic models predicted the absence of *C*. *dependens* in south-western Nigeria, which is in line with low haplotype diversity suggesting later colonization. However, given that endemic haplotypes indicate the presence of LGM refugia, it is noteworthy that the population NG02 consists of approximately 95% of the derived endemic haplotype H4. Interestingly, paleodistribution modelling revealed similar occurrence probabilities in south-eastern Nigeria as recovered for the distribution westwards from CVL during HCO using both models (Fig 3C and 3D) and during LGM using the MPI ESM-P model (Fig 3F). This finding suggests a presence of a LGM refugium of particular *C*. *dependens* lineages also in evergreen and semi-deciduous rain forest, which is in line with the recognition of several genepools of evergreen forest tree species *Erythrophleum ivorense* (Fabaceae) [13] in this area.

## Conclusions

Results from this study show that past historical factors played an important role in shaping the distribution of *Chasmanthera dependens* across West Africa. Cameroon Volcanic Line seems to represent a barrier for gene flow in the present as well as in the past, and a uniform gene flow across Nigeria and the Dahomey Gap was observed. Distribution modelling proposed refugia in the Dahomey Gap, supported also by higher genetic diversity and the presence of the derived endemic haplotype H1. This is in contrast to the phylogeographic patterns observed in several tree species and could be explained by either diverging or more relaxed ecological requirements of this liana species.

## Supporting information

S1 Table List of *Chasmanthera dependens* samples and experiments. Country codes follow ISO 3166–1 Alpha-3. UNN—University of Nigeria, Nuskka, rd–road. Collector abbreviation: CAI–Chibuzor Andrew Iloh.
(DOC)

S2 Table Geographic coordinates (longitude, latitude) used for distribution modelling. (DOC)

S3 Table Correlation coefficients between 19 climatic variables extracted for *Chasmanthera dependens*. Bold font highlights the absolute values greater than 0.8.
(DOC)

S4 Table AFLP data matrix. Ind–sample number, Pop–population. Numbers in the first line indicate the size of the fragments [bp].
(XLSX)
