# Supplementary material for: Pleistocene refugia and genetic diversity patterns in West Africa: Insights from the liana Chasmanthera dependens (Menispermaceae)
Source: PLoS One. 2017 Mar 16;12(3):e0170511. doi: 10.1371/journal.pone.0170511 (PMC5354259; doi:10.1371/journal.pone.0170511)
Supplement: S1 Table — Country codes follow ISO 3166–1 Alpha-3. UNN—University of Nigeria, Nuskka, rd–road. Collector abbreviation: CAI–Chibuzor Andrew Iloh. (DOC) [file pone.0170511.s001.doc]

S1 Table. List of *Chasmanthera dependens* samples and experiments. Country codes follow ISO 3166-1 Alpha-3. UNN - University of Nigeria, Nuskka, rd – road. Collector abbreviation: CAI – Chibuzor Andrew Iloh.

| **Sample Nr** | **PopID** | **Locality** | **AFLP** | **Haplotype** | **GenBank No.** | **Herbarium No.** |
| --- | --- | --- | --- | --- | --- | --- |
| BN/FL/017 (CAI 203)  BN/FL/018 (CAI 204)  BN/FL/020 (CAI 206)  BN/FL/021 (CAI 207)  BN/FL/026 (CAI 228)  BN/FL/029 (CAI 231)  BN/FL/030 (CAI 232)  BN/FL/031 (CAI 233)  BN/FL/034 (CAI 236)  BN/FL/035 (CAI 237)  BN/FL/042 (CAI 214)  BN/FL/044 (CAI 216)  BN/FL/045 (CAI 217)  BN/FL/046 (CAI 218)  BN/FL/048 (CAI 220)  BN/FL/050 (CAI 222)  BN/FL/052 (CAI 224)  BN/FL/053 (CAI 225)  BN/FL/055 (CAI 227)  BN/FL/056 (CAI 178)  BN/FL/058 (CAI 180)  BN/FL/062 (CAI 184)  BN/FL/063 (CAI 185) | PopBN01  PopBN01  PopBN01  PopBN01  PopBN01  PopBN01  PopBN01  PopBN01  PopBN01  PopBN01  PopBN01  PopBN01  PopBN01  PopBN01  PopBN01  PopBN01  PopBN01  PopBN01  PopBN01  PopBN01  PopBN01  PopBN01  PopBN01 | BEN; Lama Forest; CAI  BEN; Lama Forest; CAI  BEN; Lama Forest; CAI  BEN; Lama Forest; CAI  BEN; Lama Forest; CAI  BEN; Lama Forest; CAI  BEN; Lama Forest; CAI  BEN; Lama Forest; CAI  BEN; Lama Forest; CAI  BEN; Lama Forest; CAI  BEN; Lama Forest; CAI  BEN; Lama Forest; CAI  BEN; Lama Forest; CAI  BEN; Lama Forest; CAI  BEN; Lama Forest; CAI  BEN; Lama Forest; CAI  BEN; Lama Forest; CAI  BEN; Lama Forest; CAI  BEN; Lama Forest; CAI  BEN; Lama Forest; CAI  BEN; Lama Forest; CAI  BEN; Lama Forest; CAI  BEN; Lama Forest; CAI | -  -  -  -  -  -  -  -  -  -  -  -  -  -  -  -  -  -  -  -  -  -  - | H2  H2  H2  H2  H2  H2  H2  H2  H2  H2  H1  H1  H1  H2  H2  H2  H2  H2  H2  H1  H1  H1  H1 | KX863474  KX863475  KX863476  KX863477  KX863487  KX863488  KX863489  KX863490  KX863491  KX863492  KX863478  KX863479  KX863480  KX863481  KX863482  KX863483  KX863484  KX863485  KX863486  KX863470  KX863471  KX863472  KX863473 | FR-0110393  FR-0110394  FR-0110392  FR-0110390  FR-0110391  -  -  -  -  -  -  -  -  -  -  -  -  -  -  -  -  -  - |
| CMR/NG/003 (CAI 038)  CMR/NG/004 (CAI 034)  CMR/NG/005 (CAI 054)  CMR/NG/007 (CAI 056)  CMR/NG/008 (CAI 061)  CMR/NG/010 (CAI 024)  CMR/NG/011 (CAI 071)  CMR/NG/012 (CAI 050)  CMR/NG/013 (CAI 060)  CMR/NG/016 (CAI 070)  CMR/NG/018 (CAI 011)  CMR/NG/019 (CAI 062)  CMR/NG/020 (CAI 053) | PopCMR01  PopCMR01  PopCMR01  PopCMR01  PopCMR01  PopCMR01  PopCMR01  PopCMR01  PopCMR01  PopCMR01  PopCMR01  PopCMR01  PopCMR01 | CMR; 20 km NW from Bafia, towards Ngoro, along rd D50; CAI  CMR; 20 km NW from Bafia, towards Ngoro, along rd D50; CAI  CMR; 20 km NW from Bafia, towards Ngoro, along rd D50; CAI  CMR; 20 km NW from Bafia, towards Ngoro, along rd D50; CAI  CMR; 20 km NW from Bafia, towards Ngoro, along rd D50; CAI  CMR; 20 km NW from Bafia, towards Ngoro, along rd D50; CAI  CMR; 20 km NW from Bafia, towards Ngoro, along rd D50; CAI  CMR; 20 km NW from Bafia, towards Ngoro, along rd D50; CAI  CMR; 20 km NW from Bafia, towards Ngoro, along rd D50; CAI  CMR; 20 km NW from Bafia, towards Ngoro, along rd D50; CAI  CMR; 20 km NW from Bafia, towards Ngoro, along rd D50; CAI  CMR; 20 km NW from Bafia, towards Ngoro, along rd D50; CAI  CMR; 20 km NW from Bafia, towards Ngoro, along rd D50; CAI | X  X  X  -  X  X  X  X  X  X  X  X  X | H6  H6  H6  H3  H6  H6  H6  H3  H6  H3  H6  H6  H6 | KX863389  KX863385  KX863402  KX863403  KX863406  KX863376  KX863413  KX863399  KX863405  KX863412  KX863364  KX863407  KX863401 | FR-0110365  -  -  -  -  -  -  -  -  -  -  -  - |
| CMR/MF/001 (CAI 015)  CMR/MF/003 (CAI 002)  CMR/MF/004 (CAI 039)  CMR/MF/005 (CAI 006)  CMR/MF/006 (CAI 023)  CMR/MF/007 (CAI 018)  CMR/MF/008 (CAI 014)  CMR/MF/009 (CAI 032)  CMR/MF/010 (CAI 048) | PopCMR02  PopCMR02  PopCMR02  PopCMR02  PopCMR02  PopCMR02  PopCMR02  PopCMR02  PopCMR02 | CMR; Town Yaoundé, Mount Febe; CAI  CMR; Town Yaoundé, Mount Febe; CAI  CMR; Town Yaoundé, Mount Febe; CAI  CMR; Town Yaoundé, Mount Febe; CAI  CMR; Town Yaoundé, Mount Febe; CAI  CMR; Town Yaoundé, Mount Febe; CAI  CMR Town Yaoundé, Mount Febe; CAI  CMR; Town Yaoundé, Mount Febe; CAI  CMR; Town Yaoundé, Mount Febe; CAI | X  X  X  -  -  X  X  X  X | H7  H7  H3  H6  H6  H6  H6  H6  H6 | KX863368  KX863355  KX863390  KX863359  KX863375  KX863371  KX863367  KX863384  KX863398 | FR-0110378  -  -  -  -  -  -  -  - |
| GH/SB/001 (CAI 009)  GH/SB/002 (CAI 035)  GH/SB/003 (CAI 063)  GH/SB/004 (CAI 046)  GH/SB/005 (CAI 047)  GH/SB/006 (CAI 004)  GH/SB/007 (CAI 068)  GH/SB/008 (CAI 057)  GH/WR/001-1 (CAI 005)  GH/WR/001-2 (CAI 016) | PopGH01  PopGH01  PopGH01  PopGH01  PopGH01  PopGH01  PopGH01  PopGH01  PopGH01  PopGH01 | GHA; Village Seya Breku; CAI  GHA; Village Seya Breku; CAI  GHA; Village Seya Breku; CAI  GHA; Village Seya Breku; CAI  GHA; Village Seya Breku; CAI  GHA; Village Seya Breku; CAI  GHA; Village Seya Breku; CAI  GHA; Village Seya Breku; CAI  GHA; Town Winniba; CAI  GHA; Town Winniba; CAI | X  X  -  X  X  X  X  X  X  X | H2  H2  H2  H2  H2  H2  H2  H2  H2  H2 | KX863362  KX863386  KX863408  KX863396  KX863397  KX863357  KX863410  KX863404  KX863358  KX863369 | FR-0110374  -  -  -  -  -  -  -  -  - |
| NK/AB/001 (CAI 030)  NK/AB/002 (CAI 025)  NK/AB/003 (CAI 042)  NK/ABA/001 (CAI 001)  NK/ABA/002 (CAI 026)  NK/ABA/004 (CAI 043)  NK/AM/001 (CAI 010)  NK/AM/002 (CAI 044)  NK/EB/001-1 (CAI 028)  NK/EB/001-2 (CAI 051)  NK/IH/001 (CAI 037)  NK/IH/002 (CAI 008)  NK/IH/003 (CAI 027)  NK/NV/001 (CAI 017)  NK/NV/002 (CAI 022)  NK/NV/003 (CAI 029)  NK/RU/002 (CAI 019)  NK/RV/001 (CAI 066) | PopNG01  PopNG01  PopNG01  PopNG01  PopNG01  PopNG01  PopNG01  PopNG01  PopNG01  PopNG01  PopNG01  PopNG01  PopNG01  PopNG01  PopNG01  PopNG01  PopNG01  PopNG01 | NGA; Enugu State, Town Nuskka, 20 Km North East from UNN; CAI  NGA; Enugu State, Town Nuskka, 20 Km North East from UNN; CAI  NGA; Enugu State, Town Nuskka, 20 Km North East from UNN; CAI  NGA; Enugu State, Town Nuskka, 15 km South East from UNN; CAI  NGA; Enugu State, Town Nuskka, 15 km South East from UNN; CAI  NGA; Enugu State, Town Nuskka, 15 km South East from UNN; CAI  NGA; Enugu State, Town Nuskka, Village Amanzie; CAI  NGA; Enugu State, Town Nuskka, Village Amanzie; CAI  NGA; Enugu State, Town Nuskka, Village Ebu; CAI  NGA; Enugu State, Town Nuskka, Village Ebu; CAI  NGA; Enugu State, Town Nuskka, Village Ihealumuna; CAI  NGA; Enugu State, Town Nuskka, Village Ihealumuna; CAI  NGA; Enugu State, Town Nuskka, Village Ihealumuna; CAI  NGA; Enugu State, Town Nuskka, Village Ihealumuna; CAI  NGA; Enugu State, Town, Nuskka, Village Opi; CAI  NGA; Enugu State, Town, Nuskka, Village Opi; CAI  NGA; Enugu State, Town, Nuskka 5km North of Opi; CAI  NGA; Enugu State, Town, Nuskka 6km North of Opi; CAI | -  X  X  X  X  X  X  X  -  X  X  X  X  X  X  X  X  X | H3  H3  H3  H3  H3  H3  H3  H3  H3  H3  H3  H3  H3  H3  H3  H3  H3  H3 | KX863382  KX863377  KX863393  KX863354  KX863378  KX863394  KX863363  KX863395  KX863380  KX863400  KX863388  KX863361  KX863379  KX863370  KX863374  KX863381  KX863372  KX863409 | FR-0110368  FR-0110377  FR-0110370  FR-0110382  FR-0110366  FR-0110371  FR-0110367  FR-0110369  FR-0110372  -  -  -  -  -  -  -  -  - |
| NG/OB/004 (CAI 121)  NG/OB/006 (CAI 122)  NG/OB/007 (CAI 123)  NG/OB/008 (CAI 124)  NG/OB/009 (CAI 125)  NG/OB/010 (CAI 126)  NG/OB/014 (CAI 109)  NG/OB/017 (CAI 112)  NG/OB/021 (CAI 139)  NG/OB/022 (CAI 140)  NG/OB/023 (CAI 141)  NG/OB/024 (CAI 142)  NG/OB/027 (CAI 145)  NG/OB/027 (CAI 146)  NG/OB/028 (CAI 147)  NG/OB/029 (CAI 148)  NG/OB/030 (CAI 149)  NG/OB/031 (CAI 128)  NG/OB/032 (CAI 129)  NG/OB/033 (CAI 130)  NG/OB/034 (CAI 131)  NG/OB/035 (CAI 133)  NG/OB/036 (CAI 134)  NG/OB/037 (CAI 135)  NG/OB/038 (CAI 136)  NG/OB/039 (CAI 137)  NG/OB/040 (CAI 138)  NG/OB/041 (CAI 73)  NG/OB/042 (CAI 75)  NG/OB/044 (CAI 77)  NG/OB/049 (CAI 82)  NG/OB/052 (CAI 85)  NG/OB/053(CAI 86)  NG/OB/054 (CAI 87)  NG/OB/055 (CAI 88)  NG/OB/056 (CAI 89)  NG/OB/060 (CAI 94)  NG/OB/065 (CAI 101)  NG/OB/067 (CAI 99) | PopNG02  PopNG02  PopNG02  PopNG02  PopNG02  PopNG02  PopNG02  PopNG02  PopNG02  PopNG02  PopNG02  PopNG02  PopNG02  PopNG02  PopNG02  PopNG02  PopNG02  PopNG02  PopNG02  PopNG02  PopNG02  PopNG02  PopNG02  PopNG02  PopNG02  PopNG02  PopNG02  PopNG02  PopNG02  PopNG02  PopNG02  PopNG02  PopNG02  PopNG02  PopNG02  PopNG02  PopNG02  PopNG02  PopNG02 | NGA; Imo State, Town, Obinze Village Umuanunu; CAI  NGA; Imo State, Town, Obinze Village Umuanunu; CAI  NGA; Imo State, Town, Obinze Village Umuanunu; CAI  NGA; Imo State, Town, Obinze Village Umuanunu; CAI  NGA; Imo State, Town, Obinze Village Umuanunu; CAI  NGA; Imo State, Town, Obinze Village Umuanunu; CAI  NGA Imo State, Town, Obinze Village Umuanunu; CAI  NGA; Imo State, Town, Obinze Village Umuanunu; CAI  NGA; Imo State, Town, Obinze Village Umuanunu; CAI  NGA; Imo State, Town, Obinze Village Umuanunu; CAI  NGA; Imo State, Town, Obinze Village Umuanunu; CAI  NGA; Imo State, Town, Obinze Village Umuanunu; CAI  NGA; Imo State, Town, Obinze Village Umuanunu; CAI  NGA; Imo State, Town, Obinze Village Umuanunu; CAI  NGA; Imo State, Town, Obinze Village Umuanunu; CAI  NGA; Imo State, Town, Obinze Village Umuanunu; CAI  NGA; Imo State, Town, Obinze Village Umuanunu; CAI  NGA; Imo State, Town, Obinze Village Umuanunu; CAI  NGA; Imo State, Town, Obinze Village Umuanunu; CAI  NGA; Imo State, Town, Obinze Village Umuanunu; CAI  NGA; Imo State, Town, Obinze Village Umuanunu; CAI  NGA; Imo State, Town, Obinze Village Umuanunu; CAI  NGA; Imo State, Town, Obinze Village Umuanunu; CAI  NGA; Imo State, Town, Obinze Village Umuanunu; CAI  NGA; Imo State, Town, Obinze Village Umuanunu; CAI  NGA; Imo State, Town, Obinze Village Umuanunu; CAI  NGA; Imo State, Town, Obinze Village Umuanunu; CAI  NGA; Imo State, Town, Obinze Village Umuanunu; CAI  NGA; Imo State, Town, Obinze Village Umuanunu; CAI  NGA; Imo State, Town, Obinze Village Umuanunu; CAI  NGA; Imo State, Town, Obinze Village Umuanunu; CAI  NGA; Imo State, Town, Obinze Village Umuanunu; CAI  NGA; Imo State, Town, Obinze Village Umuanunu; CAI  NGA; Imo State, Town, Obinze Village Umuanunu; CAI  NGA; Imo State, Town, Obinze Village Umuanunu; CAI  NGA; Imo State, Town, Obinze Village Umuanunu; CAI  NGA; Imo State, Town, Obinze Village Umuanunu; CAI  NGA; Imo State, Town, Obinze Village Umuanunu; CAI  NGA; Imo State, Town, Obinze Village Umuanunu; CAI | -  -  -  -  -  -  -  -  -  -  -  -  -  -  -  -  -  -  -  -  -  -  -  -  -  -  -  -  -  -  -  -  -  -  -  -  -  -  - | H4  H4  H4  H4  H4  H4  H4  H4  H4  H4  H4  H4  H4  H4  H4  H4  H4  H4  H4  H4  H4  H4  H4  H4  H4  H4  H4  H2  H2  H4  H4  H4  H4  H4  H4  H4  H4  H4  H4 | KX863429  KX863430  KX863431  KX863432  KX863433  KX863434  KX863427  KX863428  KX863445  KX863446  KX863447  KX863448  KX863449  KX863450  KX863451  KX863452  KX863453  KX863435  KX863436  KX863437  KX863438  KX863439  KX863440  KX863441  KX863442  KX863443  KX863444  KX863415  KX863416  KX863417  KX863418  KX863419  KX863420  KX863421  KX863422  KX863423  KX863424  KX863426  KX863425 | FR-0110389  FR-0110387  FR-0110385  FR-0110383  FR-0110384  -  -  -  -  -  -  -  -  -  -  -  -  -  -  -  -  -  -  -  -  -  -  -  -  -  -  -  -  -  -  -  -  -  - |
| OD/OI/002 (CAI 159)  OD/OI/003 (CAI 160)  OS/OLI/001 (CAI 150)  OS/OLIII/001 (CAI 152)  OS/OLIII/002 (CAI 153)  OS/OLIV/001 (CAI 154)  OS/OLIV/002 (CAI 155) | PopNG03  PopNG03  PopNG03  PopNG03  PopNG03  PopNG03  PopNG03 | NGA; Ondo State, Okeigbo village; CAI  NGA; Ondo State, Okeigbo village; CAI  NGA; Ondo State, Okeigbo village; CAI  NGA; Osun State, Olusoye village; CAI  NGA; Osun State, Olusoye village; CAI  NGA; Osun State, Omashebi village; CAI  NGA; Osun State, Omashebi village; CAI | -  -  -  -  -  -  - | H5  H5  H5  H3  H3  H5  H5 | KX863459  KX863460  KX863454  KX863455  KX863456  KX863457  KX863458 | FR-0110386  -  -  -  -  -  - |
| IB/AY/003 (CAI 165)  IB/AY/004 (CAI 166)  IB/AY/005 (CAI 167)  IB/MR/004 (CAI 171)  IB/MR/006 (CAI 173)  IB/MR/007 (CAI 174)  IB/MR/008 (CAI 175)  IB/MR/009 (CAI 176)  IB/MR/010 (CAI 177) | PopNG04  PopNG04  PopNG04  PopNG04  PopNG04  PopNG04  PopNG04  PopNG04  PopNG04 | NGA; Oyo State, Ibadan, Adegbayi; CAI  NGA; Oyo State, Ibadan, Adegbayi; CAI  NGA; Oyo State, Ibadan, Adegbayi; CAI  NGA; Oyo State, Ibadan, Moor Plantation; CAI  NGA; Oyo State, Ibadan, Moor Plantation; CAI  NGA; Oyo State, Ibadan, Moor Plantation; CAI  NGA; Oyo State, Ibadan, Moor Plantation; CAI  NGA; Oyo State, Ibadan, Moor Plantation; CAI  NGA; Oyo State, Ibadan, Moor Plantation; CAI | -  -  -  -  -  -  -  -  - | H5  H5  H5  H5  H5  H5  H5  H5  H5 | KX863461  KX863462  KX863463  KX863464  KX863465  KX863466  KX863467  KX863468  KX863469 | FR-0110388  -  -  -  -  -  -  -  - |
| TG/AK/001 (CAI 012)  TG/AK/002 (CAI 031)  TG/AK/003 (CAI 013)  TG/AN/001 (CAI 007)  TG/AN/002 (CAI 040)  TG/AN/003 (CAI 041)  TG/AN/004 (CAI 072)  TG/AS/001 (CAI 036)  TG/AS/003 (CAI 021)  TG/AS/004 (CAI 069)  TG/TA/002 (CAI 003) | PopTG01  PopTG01  PopTG01  PopTG01  PopTG01  PopTG01  PopTG01  PopTG01  PopTG01  PopTG01  PopTG01 | TGO; Village Akoenou; CAI  TGO; Village Akoenou; CAI  TGO; Village Akoenou; CAI  TGO; Anagali Forest; CAI  TGO; Anagali Forest; CAI  TGO; Anagali Forest; CAI  TGO; Anagali Forest; CAI  TGO; Village Assome; CAI  TGO; Village Assome; CAI  TGO; Village Assome; CAI  TGO; Village Tabligbo; CAI | X  X  X  X  X  X  X  -  X  X  X | H5  H5  H2  H2  H2  H3  H2  H2  H2  H2  H5 | KX863365  KX863383  KX863366  KX863360  KX863391  KX863392  KX863414  KX863387  KX863373  KX863411  KX863356 | FR-0110381  FR-0110379  FR-0110380  FR-0110373  FR-0110376  -  -  -  -  -  - |
