# Supplementary material for: Pleistocene refugia and genetic diversity patterns in West Africa: Insights from the liana Chasmanthera dependens (Menispermaceae)
Source: PLoS One. 2017 Mar 16;12(3):e0170511. doi: 10.1371/journal.pone.0170511 (PMC5354259; doi:10.1371/journal.pone.0170511)
Supplement: S2 Table — (DOC) [file pone.0170511.s002.doc]

| **Longitude** | **Latitude** |
| --- | --- |
| -13.25 | 8.5 |
| -13.25 | 8.5 |
| -11.42 | 8.42 |
| -5.51667 | 7.68333 |
| -5.1656 | 8.80907 |
| -4.9 | 6.4 |
| -4.87 | 5.98 |
| -4.65 | 9.75 |
| -4.56667 | 5.63333 |
| -4.13333 | 5.31667 |
| -3.9 | 5.33333 |
| -2.15 | 7.3 |
| -2.10167 | 7.775 |
| -2.10166667 | 7.775 |
| -2.061 | 7.465 |
| -2 | 7 |
| -1.66 | 5.65 |
| -1.5 | 5.31 |
| -1.27 | 6.06 |
| -1.16 | 5.08 |
| -0.54555 | 5.49362 |
| -0.53417 | 5.48722 |
| -0.5 | 6.05 |
| -0.33 | 6.1 |
| -0.28 | 7.32 |
| -0.14 | 5.37 |
| -0.03 | 7.47 |
| 0.31 | 8.16 |
| 0.35 | 8.19 |
| 0.51666667 | 8.26666667 |
| 0.51667 | 8.26667 |
| 0.58333333 | 8.31666667 |
| 1.15222 | 6.36138 |
| 1.34 | 6.65 |
| 1.35805 | 6.51722 |
| 1.36112 | 6.51028 |
| 1.36278 | 6.58862 |
| 1.41667 | 7.43333 |
| 1.59888 | 6.24555 |
| 1.712 | 85.195 |
| 1.733 | 7.35 |
| 2.01011 | 6.44376 |
| 2.0833 | 6.85 |
| 2.11445 | 6.97055 |
| 2.11472 | 6.97612 |
| 2.1325 | 6.96972 |
| 2.13278 | 6.97 |
| 2.13305 | 6.97388 |
| 2.183 | 7.833 |
| 2.18333 | 7.83333 |
| 2.18333333 | 7.83333333 |
| 2.2 | 7.833 |
| 2.2 | 7.83333 |
| 2.2 | 7.83333333 |
| 2.3 | 7.2333 |
| 2.33333 | 8.26667 |
| 2.33333333 | 8.26666667 |
| 2.366 | 7.433 |
| 2.36666667 | 7.43333333 |
| 2.36667 | 7.43333 |
| 2.3833 | 10.35 |
| 2.4 | 7.43333 |
| 2.4 | 7.43333333 |
| 2.4333 | 6.35 |
| 2.45 | 6.9 |
| 2.45 | 6.983 |
| 2.45 | 6.98333 |
| 2.45 | 6.98333333 |
| 2.4666 | 7.0166 |
| 2.53333333 | 6.51666667 |
| 2.628 | 6.515 |
| 2.6833 | 6.9666 |
| 2.68333 | 6.96667 |
| 2.68333333 | 6.96666667 |
| 2.73384 | 6.68334 |
| 3.33 | 7.09 |
| 3.36667 | 7.41667 |
| 6.62061 | 0.25376 |
| 6.96778 | 5.40333 |
| 6.96778 | 5.40362 |
| 6.96805 | 5.40305 |
| 6.96862 | 5.40417 |
| 6.99 | 5.87 |
| 7.41278 | 6.80862 |
| 7.42445 | 6.83612 |
| 7.44278 | 6.83945 |
| 7.45917 | 6.84195 |
| 7.45945 | 6.84195 |
| 7.46083 | 6.84083 |
| 7.46167 | 6.80612 |
| 7.46167 | 6.84333 |
| 7.46222 | 6.84305 |
| 7.99 | 9.31 |
| 8.58333333 | 3.48333333 |
| 8.69149 | 3.49651 |
| 8.78 | 3.74916667 |
| 8.78333333 | 3.76666667 |
| 10.5 | 9.75 |
| 11.35083 | 4.87805 |
| 11.43 | 4.54 |
| 11.4833333 | 3.86666667 |
| 11.4833333 | 3.91666667 |
| 11.49417 | 3.915 |
| 16.53 | 3.76 |
| 18.31 | 10.31 |
| 20.08 | 8.2 |
| 20.53 | 5.87 |
| 23.96 | 13.97 |
| 29.51 | 2.21 |
| 30.73 | -10.32 |
| 30.73 | -6.33 |
| 32.28 | 5.32 |
| 32.62 | 0.55 |
| 32.91667 | -8.35 |
| 33.25 | -8.78333 |
| 33.80028 | -13.9425 |
| 35.83 | 3.65 |
| 36.7166667 | 5.85 |
| 36.76095 | 5.720392 |
| 36.88277 | -7.83444 |
| 37.55 | 5.23333 |
| 37.55 | 5.23333333 |
| 37.86667 | -0.41667 |
| 38.94 | 8.98 |
| 39.16 | 2.77 |
| 39.38 | -1.34 |
| 40.0333333 | 8.83333333 |
| 40.27 | 14.86 |
| 42.27 | 2.88 |
| 42.71 | 1.1 |
| 45.7 | 3.21 |
