# Supplementary material for: Pleistocene refugia and genetic diversity patterns in West Africa: Insights from the liana Chasmanthera dependens (Menispermaceae)
Source: PLoS One. 2017 Mar 16;12(3):e0170511. doi: 10.1371/journal.pone.0170511 (PMC5354259; doi:10.1371/journal.pone.0170511)
Supplement: S3 Table — Bold font highlights the absolute values greater than 0.8. (DOC) [file pone.0170511.s003.doc]

S3 Table. Correlation coefficients between 19 climatic variables extracted for the *Chasmanthera dependens*. Red font highlights the absolute values greater than 0.8. Variables in the first line of the table indicated by red color were excluded due to high correlation with other variables.

|  | bio_1 | bio_2 | bio_3 | bio_4 | bio_5 | bio_6 | bio_7 | bio_8 | bio_9 | bio_10 | bio_11 | bio_12 | bio_13 | bio_14 | bio_15 | bio_16 | bio_17 | bio_18 | bio_19 |
| --- | --- | --- | --- | --- | --- | --- | --- | --- | --- | --- | --- | --- | --- | --- | --- | --- | --- | --- | --- |
| bio_1 | 1.00 | 0.13 | -0.27 | 0.31 | 0.85 | 0.57 | 0.25 | 0.85 | 0.83 | 0.93 | 0.78 | -0.33 | -0.25 | -0.14 | 0.39 | -0.30 | -0.15 | -0.44 | 0.01 |
| bio_2 | 0.13 | 1.00 | -0.46 | 0.58 | 0.58 | -0.63 | 0.88 | 0.28 | -0.19 | 0.33 | -0.24 | -0.58 | -0.41 | -0.43 | 0.67 | -0.41 | -0.49 | -0.55 | -0.36 |
| bio_3 | -0.27 | -0.46 | 1.00 | -0.87 | -0.61 | 0.47 | -0.80 | -0.45 | 0.08 | -0.55 | 0.28 | 0.63 | 0.39 | 0.61 | -0.72 | 0.39 | 0.66 | 0.53 | 0.41 |
| bio_4 | 0.31 | 0.58 | -0.87 | 1.00 | 0.68 | -0.50 | 0.87 | 0.58 | -0.09 | 0.62 | -0.35 | -0.73 | -0.60 | -0.42 | 0.74 | -0.61 | -0.48 | -0.56 | -0.41 |
| bio_5 | 0.85 | 0.58 | -0.61 | 0.68 | 1.00 | 0.08 | 0.71 | 0.84 | 0.53 | 0.95 | 0.40 | -0.61 | -0.45 | -0.38 | 0.69 | -0.48 | -0.42 | -0.69 | -0.18 |
| bio_6 | 0.57 | -0.63 | 0.47 | -0.50 | 0.08 | 1.00 | -0.65 | 0.26 | 0.80 | 0.28 | 0.88 | 0.33 | 0.22 | 0.34 | -0.38 | 0.19 | 0.38 | 0.14 | 0.43 |
| bio_7 | 0.25 | 0.88 | -0.80 | 0.87 | 0.71 | -0.65 | 1.00 | 0.46 | -0.16 | 0.53 | -0.31 | -0.70 | -0.50 | -0.53 | 0.80 | -0.50 | -0.59 | -0.63 | -0.44 |
| bio_8 | 0.85 | 0.28 | -0.45 | 0.58 | 0.84 | 0.26 | 0.46 | 1.00 | 0.49 | 0.89 | 0.44 | -0.52 | -0.46 | -0.20 | 0.57 | -0.50 | -0.23 | -0.44 | -0.22 |
| bio_9 | 0.83 | -0.19 | 0.08 | -0.09 | 0.53 | 0.80 | -0.16 | 0.49 | 1.00 | 0.66 | 0.88 | -0.03 | -0.04 | 0.07 | -0.03 | -0.07 | 0.08 | -0.26 | 0.28 |
| bio_10 | 0.93 | 0.33 | -0.55 | 0.62 | 0.95 | 0.28 | 0.53 | 0.89 | 0.66 | 1.00 | 0.52 | -0.54 | -0.42 | -0.28 | 0.59 | -0.46 | -0.31 | -0.59 | -0.13 |
| bio_11 | 0.78 | -0.24 | 0.28 | -0.35 | 0.40 | 0.88 | -0.31 | 0.44 | 0.88 | 0.52 | 1.00 | 0.15 | 0.15 | 0.13 | -0.10 | 0.11 | 0.15 | -0.09 | 0.30 |
| bio_12 | -0.33 | -0.58 | 0.63 | -0.73 | -0.61 | 0.33 | -0.70 | -0.52 | -0.03 | -0.54 | 0.15 | 1.00 | 0.88 | 0.56 | -0.71 | 0.90 | 0.61 | 0.76 | 0.60 |
| bio_13 | -0.25 | -0.41 | 0.39 | -0.60 | -0.45 | 0.22 | -0.50 | -0.46 | -0.04 | -0.42 | 0.15 | 0.88 | 1.00 | 0.26 | -0.41 | 0.98 | 0.30 | 0.61 | 0.51 |
| bio_14 | -0.14 | -0.43 | 0.61 | -0.42 | -0.38 | 0.34 | -0.53 | -0.20 | 0.07 | -0.28 | 0.13 | 0.56 | 0.26 | 1.00 | -0.60 | 0.28 | 0.98 | 0.50 | 0.39 |
| bio_15 | 0.39 | 0.67 | -0.72 | 0.74 | 0.69 | -0.38 | 0.80 | 0.57 | -0.03 | 0.59 | -0.10 | -0.71 | -0.41 | -0.60 | 1.00 | -0.46 | -0.66 | -0.60 | -0.43 |
| bio_16 | -0.30 | -0.41 | 0.39 | -0.61 | -0.48 | 0.19 | -0.50 | -0.50 | -0.07 | -0.46 | 0.11 | 0.90 | 0.98 | 0.28 | -0.46 | 1.00 | 0.32 | 0.64 | 0.53 |
| bio_17 | -0.15 | -0.49 | 0.66 | -0.48 | -0.42 | 0.38 | -0.59 | -0.23 | 0.08 | -0.31 | 0.15 | 0.61 | 0.30 | 0.98 | -0.66 | 0.32 | 1.00 | 0.54 | 0.42 |
| bio_18 | -0.44 | -0.55 | 0.53 | -0.56 | -0.69 | 0.14 | -0.63 | -0.44 | -0.26 | -0.59 | -0.09 | 0.76 | 0.61 | 0.50 | -0.60 | 0.64 | 0.54 | 1.00 | 0.19 |
| bio_19 | 0.01 | -0.36 | 0.41 | -0.41 | -0.18 | 0.43 | -0.44 | -0.22 | 0.28 | -0.13 | 0.30 | 0.60 | 0.51 | 0.39 | -0.43 | 0.53 | 0.42 | 0.19 | 1.00 |

bio_1 = Annual Mean Temperature bio_13 = Precipitation of Wettest Month

bio_2 = Mean Diurnal Range (Mean of monthly (max temp - min temp)) bio_14 = Precipitation of Driest Month

bio_3 = Isothermality (bio_2/bio_7) (* 100) bio_15 = Precipitation Seasonality (Coefficient of Variation)

bio_4 = Temperature Seasonality (standard deviation *100) bio_16 = Precipitation of Wettest Quarter

bio_5 = Max Temperature of Warmest Month bio_17 = Precipitation of Driest Quarter

bio_6 = Min Temperature of Coldest Month bio_18 = Precipitation of Warmest Quarter

bio_7 = Temperature Annual Range (bio_5-bio_6) bio_19 = Precipitation of Coldest Quarter

bio_8 = Mean Temperature of Wettest Quarter

bio_9 = Mean Temperature of Driest Quarter

bio_10 = Mean Temperature of Warmest Quarter

bio_11 = Mean Temperature of Coldest Quarter

bio_12 = Annual Precipitation
